# Mitochondrial extracellular vesicles, autoimmunity and myocarditis

**DOI:** 10.3389/fimmu.2024.1374796

**Published:** 2024-03-14

**Authors:** Damian N. Di Florio, Danielle J. Beetler, Elizabeth J. McCabe, Jon Sin, Tsuneya Ikezu, DeLisa Fairweather

**Affiliations:** ^1^ Department of Cardiovascular Medicine, Mayo Clinic, Jacksonville, FL, United States; ^2^ Center for Clinical and Translational Science, Mayo Clinic, Rochester, MN, United States; ^3^ Mayo Clinic Graduate School of Biomedical Sciences, Mayo Clinic, Rochester, MN, United States; ^4^ Department of Biological Sciences, University of Alabama, Tuscaloosa, AL, United States; ^5^ Department of Neuroscience, Mayo Clinic, Jacksonville, FL, United States; ^6^ Department of Immunology, Mayo Clinic, Jacksonville, FL, United States; ^7^ Department of Medicine, Mayo Clinic, Jacksonville, FL, United States

**Keywords:** autoimmune disease, extracellular vesicles, mitochondria, mitochondrial-derived vesicles, myocarditis, AIRE, coxsackievirus

## Abstract

For many decades viral infections have been suspected as ‘triggers’ of autoimmune disease, but mechanisms for how this could occur have been difficult to establish. Recent studies have shown that viral infections that are commonly associated with viral myocarditis and other autoimmune diseases such as coxsackievirus B3 (CVB3) and SARS-CoV-2 target mitochondria and are released from cells in mitochondrial vesicles that are able to activate the innate immune response. Studies have shown that Toll-like receptor (TLR)4 and the inflammasome pathway are activated by mitochondrial components. Autoreactivity against cardiac myosin and heart-specific immune responses that occur after infection with viruses where the heart is not the primary site of infection (e.g., CVB3, SARS-CoV-2) may occur because the heart has the highest density of mitochondria in the body. Evidence exists for autoantibodies against mitochondrial antigens in patients with myocarditis and dilated cardiomyopathy. Defects in tolerance mechanisms like autoimmune regulator gene (AIRE) may further increase the likelihood of autoreactivity against mitochondrial antigens leading to autoimmune disease. The focus of this review is to summarize current literature regarding the role of viral infection in the production of extracellular vesicles containing mitochondria and virus and the development of myocarditis.

## Highlights

- Mitochondrial extracellular vesicles contain CVB3
- Extracellular vesicles containing mitochondrial components activate TLR4/NLRP3
- Autoantibodies against mitochondria are found in patients with myocarditis
- The autoimmune regulator AIRE may bind few mitochondrial genes

## Introduction

The immune system protects the host against infection by specifically recognizing and eliminating foreign pathogens, but in the process must avoid responding to host antigens. During maturation of the immune system, immune cells that react against self-antigens are eliminated providing an immune system that is ‘tolerant’ to self (1). T cells that escape central tolerance are additionally regulated with peripheral tolerance mechanisms that include the conversion of self-reactive T cells to regulatory T cells. Autoimmunity that progresses to autoimmune disease can occur if this process breaks down (2). Genetic and environmental factors contribute to the development of autoimmune diseases, but twin studies indicate that environmental factors are a significant contributor (3, 4). For many decades viral infections have been suspected as ‘triggers’ of autoimmune disease, but mechanisms for how this could occur have been difficult to establish (2, 5, 6). Recent findings suggest that subversion of host cellular extracellular vesicle (EV) processing by viral infections may lead not only to activation of the immune response against the virus but also against mitochondrial or other self-antigens thereby contributing to the development of autoimmune disease. In this review, we describe EVs with mitochondrial content, their relationship to viral infections such as coxsackievirus B3 (CVB3), and their potential role in driving autoimmune diseases with a focus on myocarditis.

### Extracellular vesicles

In the last decade there has been a major increase in interest in EVs in their role in cell-to-cell communication, as biomarkers and as therapeutics (7, 8). Many terms and definitions are used to describe EVs, and in this review we use the term EVs to refer to all extracellular, lipid bilayer, sub-cellular particles and their functional contents with sizes ranging from several nm to several μm (9, 10). This umbrella term includes the widely recognized major subgroups termed exosomes, microvesicles, and apoptotic bodies, which are currently distinguishable only by their theorized origin and size but not by experimental means (11). EVs are engaged in cellular communication in both health and disease as transporters of molecular signals in the form of nucleic acids (e.g., DNA, mRNA, microRNA/miRs, long-coding RNA/lcRNA and circular RNA/circRNA), proteins and lipids (11).

When tissue environments are perturbed or cells become damaged as occurs during a viral infection, EV content changes based on cellular reprogramming in response to pathological stress (11, 12). EVs can either activate or inhibit innate and adaptive immune cell responses based on their content (13–15). EVs have been demonstrated to express major histocompatibility complex (MHC) class I or II and directly activate innate antigen presenting cells (APCs) or adaptive T and B cells in an antigen/self-antigen-specific manner (16, 17). Tetraspanins like CD9, CD63 and CD81, which are commonly used to characterize EVs, bind factors on innate immune cells like integrins (i.e., CD11b) that are important in activating and modulating immune responses (12, 18).

### Viral infection and EVs

Importantly, many viruses use EV cellular machinery (i.e., exosome endosomal sorting complexes required for the transport/ESCRT pathway) for viral transmission such as cytomegalovirus, coxsackievirus, SARS-CoV-2, human immunodeficiency virus 1 (HIV-1), hepatitis viruses B, C and E (HBV, HCV, and HEV), and multiple members of the human herpesvirus (HHV) family (reviewed in (19–22). As a result, EVs can contain infectious virus, viral particles and/or viral proteins following infection that can subvert the immune response to promote viral replication. Important from an autoimmune disease context, a ‘mix’ of self and foreign antigens in/on EVs that may occur after viral infection may be presented to APCs and drive the immune response to target not only the infectious agent but also host antigens resulting in an autoimmune response.

### EVs and autoimmune disease

The role of EVs in the development of autoimmune disease has been studied in patients and animal models. A review article by Tian et al. recently examined the role of EVs in a number of autoimmune diseases including thyroiditis, systemic lupus erythematosus (SLE), multiple sclerosis (MS), rheumatoid arthritis (RA), anti-phospholipid syndrome and type I diabetes (23). Many investigators have reported that the number of circulating EVs are elevated in patients with autoimmune disease compared to controls (24–26); however, the wide variety of methods and procedures for isolating EVs as well as differences in storage conditions makes it difficult to interpret these findings.

Studies examining changes in EV content and function may provide a clearer picture of their effects in patients with autoimmune disease. MicroRNA (miRs) content in blood EVs (exosomes) were identified as biomarkers that distinguished patients with relapsing-remitting MS (i.e., miR-15b-5p, miR-451a, miR-30b-5p, miR-342-3p) from those with progressive MS or healthy controls (27, 28). Eight out of the nine miRs that were identified in the study were confirmed in a separate group of patients indicating that the miRs/EVs could serve as biomarkers to predict MS type. Similar results have been found for other autoimmune diseases like type I diabetes (29). EVs have also been found to either promote inflammation/remodeling or to inhibit harmful immune responses for a number of autoimmune diseases including RA (30–33), Hashimoto’s thyroiditis (34), type I diabetes (35), SLE (36), and myocarditis (15). Additionally, EVs have been found with immunoglobulins (Ig) on their surface including IgG or internally in the form of self-antigen-complement-Ig immune complexes (ICs) (37, 38) suggesting that EVs may initiate and/or promote autoimmune damage and inflammation via ICs (39–42). Understanding the role of EVs in autoimmune disease is an emerging field with many questions still to be answered.

## Mitochondrial extracellular vesicles

**Figure 1:**
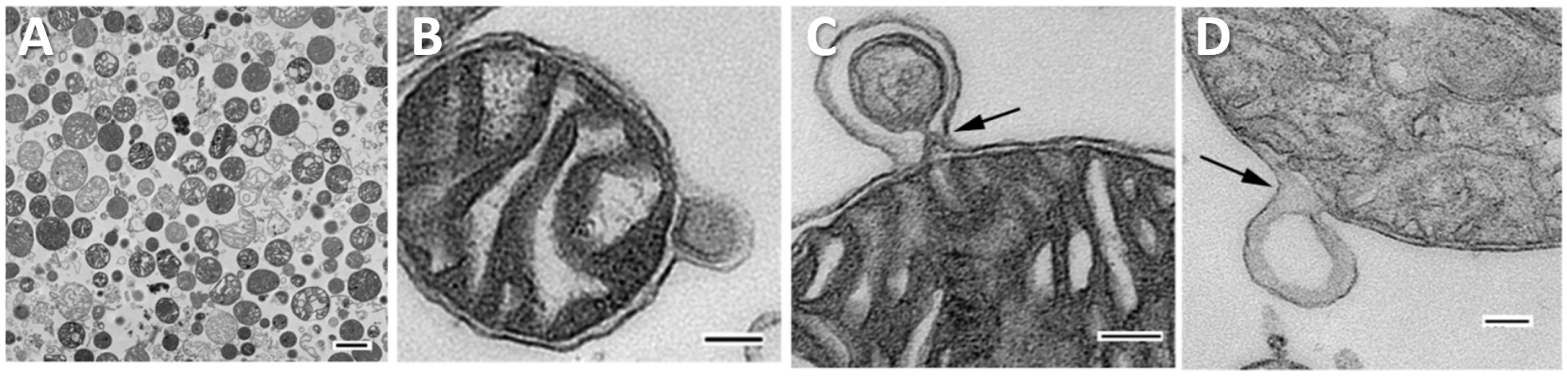
Mitochondrial derived vesicles (MDVs) from cardiac mitochondria. Widefield transmission electron microscopic images of mitochondria isolated from bovine heart. **(A)** 60-100 nm vesicles containing mitochondria. Scale bar, 500 nm, **(B)** MDV budding from mitochondria containing inner and outer mitochondrial membrane, **(C)** Protrusion of MDV from mitochondria showing constriction at its base, **(D)** MDV forming with only outer mitochondrial membrane. Panels **(B–D)**, scale bars 100 nm. Reused with permission from (54).

Another form of EVs that have received recent attention and may play a role in autoimmune disease are those that contain host cellular components such as mitochondria (e.g., primarily mitochondrial proteins or RNA) (43). The earliest evidence of mitochondria and mitochondrial components in vesicles comes from a description by Vishwa Nath in 1932 of work by Koltzoff in 1906 studying sperm cells from *Paratelphusa spinigera* (44). Koltzoff and Nath observed sub-cellular structures in crab spermatocytes undergoing a process that sounds similar to our current understanding of mitochondrial-derived vesicles (MDVs) (intracellular vesicles for mitochondrial transport) (44–46) or mitophagosomes (mitochondria fission products contained in autophagosomes for selective autophagy) (47, 48). Both MDVs and fragmented mitochondria fission products can be sent to autophagosomes for selective autophagy (46, 49) in a specific lysosomal degradation of mitochondria process referred to as ‘mitophagy’ (49). The formation of the endoplasmic reticulum barrier around fragmented mitochondrial pieces (i.e., the autophagosome), a process that occurs in receptor-mediated mitophagy, is what Nath suspected protected these structures (which he only knew as another membrane around a mitochondrial mass) from rupture and lysis when exposed to acetic acid (44). Another process that has been referred to as ‘mitoptosis’ involves selective removal of damaged mitochondria from the cell in vesicles (i.e., EVs) that are generally referred to as mitochondrial EVs (50) or mitovesicles (51) that contain whole or pieces of mitochondria (52, 53). See images of mitochondrial EVs budding from cardiac myocytes in **Figure 1** (54). Importantly, this process can occur for healthy physiological removal or transfer of mitochondria as well as for damaged mitochondria (55).

EVs that contain mitochondria lack standardized definitions but are known to contain inner and outer mitochondrial membrane components, mitochondrial nucleic acid (i.e., DNA, RNA), and/or cardiolipin - a signature phospholipid that is more concentrated in mitochondrial membranes than cellular membranes (9). The two known major populations of mitochondrial EVs also differ in terms of their size: MDVs are smaller (30-100 nm) and EVs containing larger mitochondrial components or whole mitochondria have a larger size.

### Coxsackievirus B3-induced mitochondrial EVs

**Figure 2:**
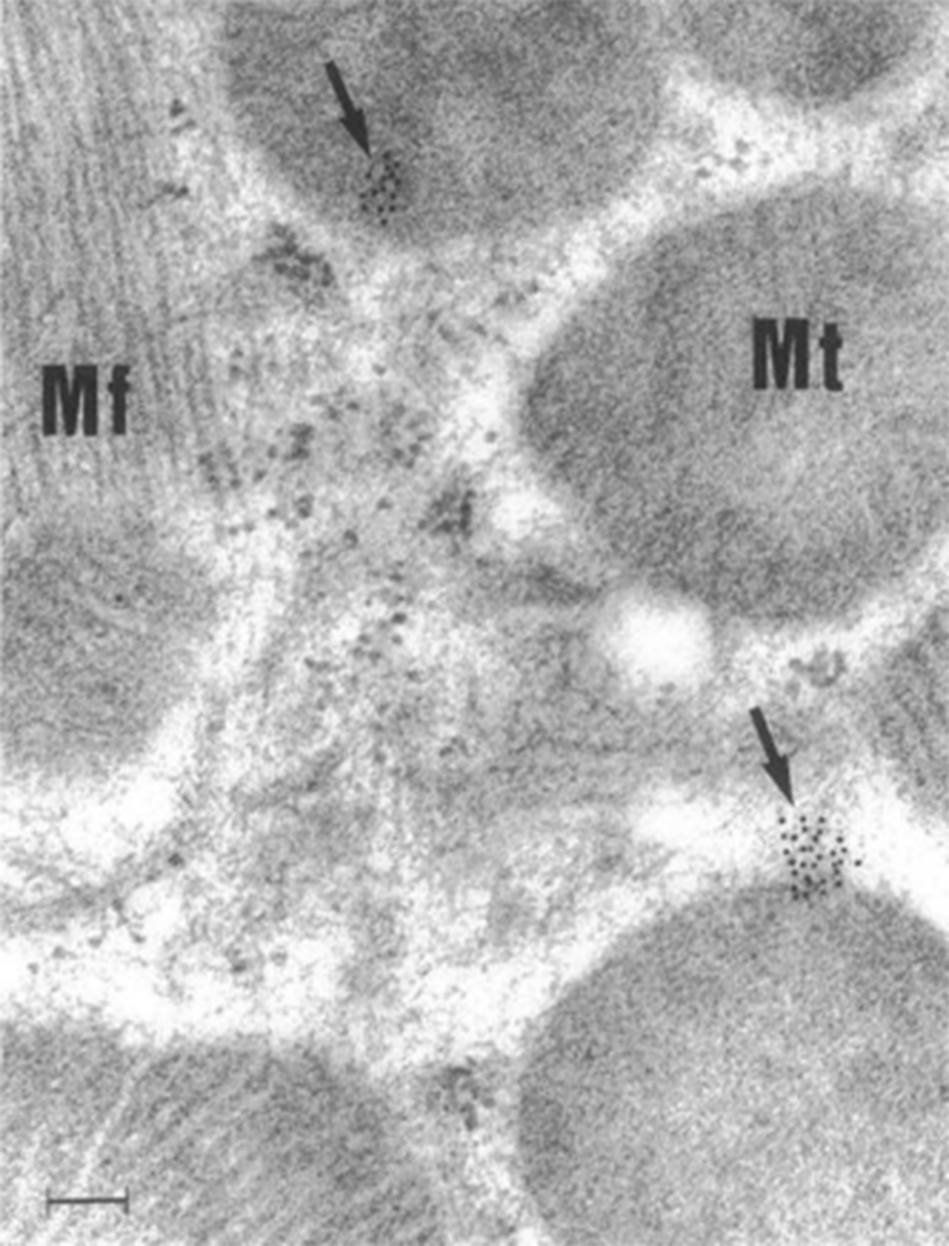
CVB3 localizes around and within murine cardiac mitochondria during myocarditis. Immunogold electron micrograph of mouse cardiomyocyte with CVB3 myocarditis on day 8 post infection. Black dots (arrows) are gold staining of CVB3 viral genome localizing around and in cardiomyocyte mitochondria. Scale bar, 100 nm. Mt, mitochondria; Mf, myofibril. Reused with permission from (58).

For decades, small non-enveloped RNA viruses like CVB3 were thought to cause host cell lysis as the primary method of viral dissemination, but recent evidence has demonstrated that infectious CVB3 and viral particles are released in mitochondrial EVs (47, 56). The first evidence that CVB3 infection disrupts cardiac mitochondria was published in 1964 using young Swiss white mice (Webster strain) inoculated with tissue culture-derived virus (57). Investigators utilized microscopy to assess subcellular changes to the myocardium during viral infection. Notably, they identified an increase in mitochondrial fission, disruption of mitochondrial cristae, and smaller mitochondria with additional membranes that enclosed them that likely depict mitophagosomes (57). A later study from 1997 identified CVB3 localization around and *within* cardiac mitochondria during myocarditis in mice (**Figure 2**) (58).

**Figure 3:**
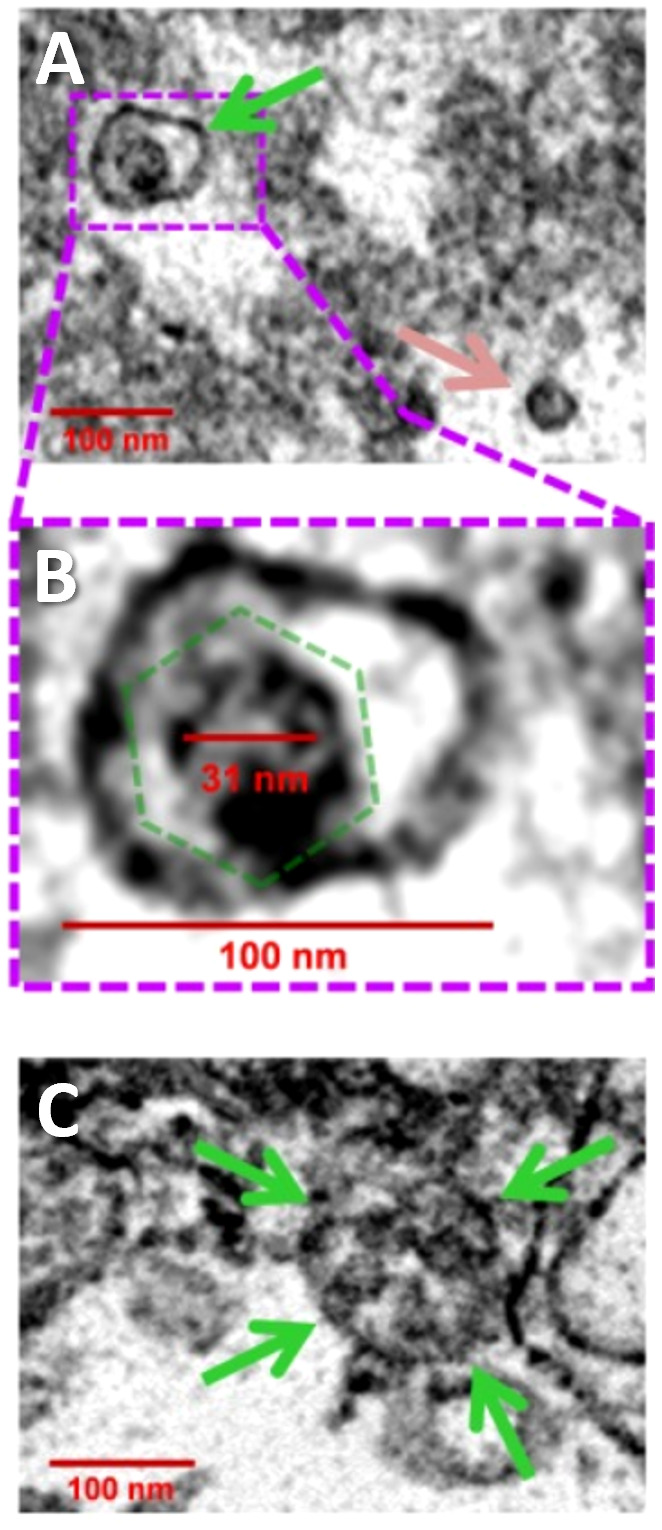
CVB3 identified in EVs using transmission electron microscopy. **(A)** Widefield transmission electron microscopic view of single virion (green arrow) in an extracellular EV or free virion (pink arrow) from culture of CVB3 in C2C12 cells. **(B)** Higher digital magnification (dashed purple box) of a virus-like particle revealed an icosahedral shape structure (dashed green polygon) slightly larger than 31 nm in diameter enclosed within a membrane structure. **(C)** Large EV containing multiple virions (green arrows). Scale bars = 100 nm. Reused with permission from (47).

In 2017, Roberta Gottlieb’s laboratory published a study demonstrating CVB3 viral transmission via EVs containing mitochondrial components (47). Using an *in vitro* neural progenitor stem cell model of viral infection, Robinson et al. demonstrated that CVB3 localizes to the mitochondrial compartment of infected cells and is later ejected from cells in vesicles containing virus, inner and outer mitochondrial membrane components, and autophagy machinery (i.e., microtubule-associated protein light chain 3/LC3-II) (47). Using electron microscopy, they found these particles ranged in size from 100-200 nm in diameter and contained single or multiple virions (**Figure 3**) (47). They also observed that the ejected particles were infectious to adjacent uninfected host cells.

**Figure 4:**
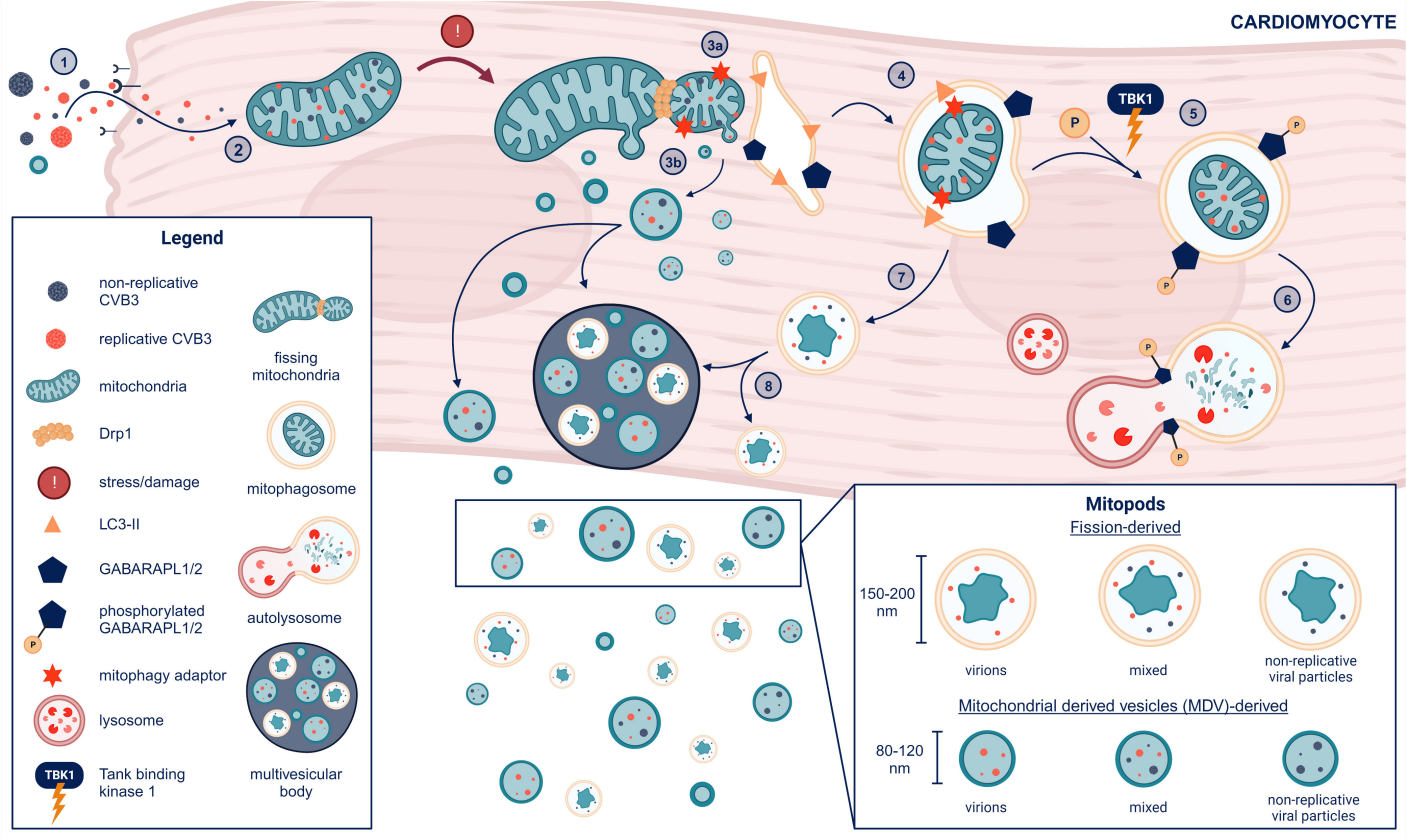
Formation of mitochondrial EVs from cardiomyocytes after CVB3 infection (1). CVB3 gains entry to cardiomyocytes via the coxsackievirus adrenoreceptor (CAR) or passive entry from previously formed mitochondrial EVs containing replicative virus (2). CVB3 mitochondrial localization induces mitochondrial stress and damage leading to (3a) mitochondrial-derived vesicle (MDV) formation and Drp1-mediated mitochondrial fission and recruitment of the endoplasmic reticulum (ER) for autophagosome formation alongside LC3 lipidation (LC3-II). (3b) MDVs containing replicative and or non-replicative viral particles may either eject from the cell or join multi-vesicular bodies before release from cardiomyocytes (MDVs can also be slated for receptor mediated mitophagy and potentially escape the cell without GABARAPL phosphorylation, which is not shown in this diagram) (4). LC3-II binds mitophagy adaptors situated on the outer mitochondrial membrane to form a mitophagosome with GABARAPL proteins on the endoplasmic reticulum (ER) facing the cytosol (5). Phosphorylation of the mitophagosome on GABARAPL proteins by tank-binding kinase 1 (TBK1) leads mitophagosomes to subsequent (6) lysosomal fusion and degradation (7). Non-phosphorylated mitophagosomes do not proceed to fusion with the lysosome but either (8) join the multivesicular body for cell release and dissemination or are ejected alone. The resulting EVs containing mitochondrial components and viral particles (replicative and/or non-replicative) we term as “mitopods” or mitochondrial escape-pods for CVB3. The two major sources of mitopods are MDV-derived or fission-derived. Another possible distinguishing feature of fission-derived versus MDV-derived mitopods would be an additional membrane derived from the ER. This figure was created using BioRender.

The protein dynamin-related protein 1 (Drp1) is required for mitochondria to undergo fission. Drs. Gottlieb and Sin showed that CVB3 infection led to Drp1-induced mitochondrial fission resulting in damaged mitochondria being processed into mitophagosomes via mitophagy and released from host HL-1 cardiomyocytes in culture as mitochondrial EVs (48). The role of fission in the production of mitochondrial vesicles was confirmed by inhibition/blocking mitochondrial fission machinery using mitochondrial division inhibitor-1 (Mdivi-1) or direct inhibition of Drp1 with siRNA which resulted in less viral replication and fewer/no virus containing EVs in the culture supernatant (48). Dr. Sin’s group additionally showed that Tank binding kinase I (TBK1) increased phosphorylation of GABA type A receptor associated protein-like (GABARAPL) proteins leading to EVs that contain mitochondria being released from the cells (59). CVB3 infection has also been shown to perturb syntaxin-17 facilitated mitophagosome-lysosomal fusion, which may lead to build up and release of formed mitophagosomes from the cell (60). Thus, these studies confirm that CVB3 localizes to mitochondria and is released in mitochondrial vesicles. Further research is needed to better understand the molecular mechanisms of intracellular mitophagosome formation in the context of viral infections to determine how viruses take advantage of mitochondrial compartments and evade intracellular degradation by targeted autophagy. A summary of our current understanding of CVB3-mitochondria interaction and the development of EV populations containing mitochondria and virus is illustrated in **Figure 4**.

## Mitochondrial autoimmunity and myocarditis

It turns out that many viruses are known to localize to mitochondria (61–64), utilize mitochondrial machinery for replication (48, 65), evade immune responses within EVs (66) and modify cellular processes (59, 60, 67). Importantly, most of the viruses that are associated with causing myocarditis [e.g., CVB, influenza, HIV, poliovirus, hepatitis C virus, SARS-CoV-22 (68, 69)] have been found to target mitochondria to gain a replicative advantage (61–64) and are ejected from cells/tissues in EVs (60, 65, 70, 71) suggesting that these mechanisms may provide an explanation for how viruses could cause autoimmune disease.

### Mitochondrial autoantibodies in patients with myocarditis

Dr. Peter Schultheiss, a major contributor to the fields of cardiology and myocarditis, began identifying and characterizing autoimmune antibodies in patients with myocarditis in the 1970s. In 1978, Bolte and Schultheiss reported that 76% of 17 patients with viral myocarditis had autoantibodies in sera and 41% of these were anti-nuclear antibodies (72). They went on to show that autoantibodies against the adenine nucleotide transporter (ANT), which is a component of the inner mitochondrial membrane, were elevated in patients with myocarditis and dilated cardiomyopathy (DCM) (73, 74). Myocarditis progresses to DCM in susceptible patients and animal models (75). Patients with suspected or confirmed viral myocarditis or cardiomyopathy/DCM had the highest reactivity to anti-mitochondrial antigen and highest expression of anti-mitochondrial antibodies (74). Further analysis found that the sera had uniquely specific reactivity toward cardiac mitochondrial antigen compared to liver or kidney mitochondrial antigen (76).

Another group independently reported that patients with various cardiomyopathies including myocarditis had autoantibodies reactive against mitochondrial proteins (77). They found that 13% with acute myocarditis, 31% of patients with DCM, and 33% with hypertrophic cardiomyopathy generated antibody responses specifically to the M7 antigen of the mitochondrial enzyme sarcosine dehydrogenase, and 25% of these reacted against the cardiac-specific form of the mitochondrial antigen (77). Another group observed autoantibodies against ANT in patients diagnosed with myocarditis or DCM (78). They also observed cardiac-specific reactivity and suggested mitochondrial autoimmune activity as a potential mechanism for the development of DCM following acute myocarditis (78). This agrees with our current understanding of the development of DCM following acute myocarditis (75, 79, 80).

### Mitochondrial autoantibodies in models of myocarditis

Although viral-induced myocarditis is often categorized as a distinct condition from autoimmune myocarditis clinically and in animal models, the distinction between the two conditions is not clear-cut because patients with viral myocarditis and mouse models of viral myocarditis have been demonstrated to develop autoantibodies and autoreactive T and B cells against cardiac myosin and other self-antigens including mitochondria (2, 39, 79, 81–83).

Importantly, a study examining autoantibody levels that compared experimental autoimmune myocarditis (EAM) to CVB3-induced myocarditis in mice found that ANT was only produced after viral infection but not in EAM suggesting that viral infection was necessary for the production of mitochondrial autoantibodies whereas both models produced autoantibodies against cardiac myosin (84). Additionally, Lin et al. showed that depletion of Drp1 (required for fission) in mice using the mitochondrial fission inhibitor Mdivi-1 reduced CVB3 myocarditis and restored mitochondrial function in the heart (71) suggesting that mitochondrial EVs containing virus may increase myocarditis, although they did not examine this in the study. Overall, these findings suggest that viral infection may be an important mechanism to produce mitochondrial autoantibodies found in patients with autoimmune diseases.

### Anti-mitochondrial antibodies in rheumatic autoimmune diseases

Anti-mitochondrial antibodies (for example, antibodies that target cardiolipin, mitofusin 1, mitochondrial DNA or mitochondrial RNA) are commonly found in patients with rheumatic autoimmune diseases such as RA, SLE, and anti-phospholipid syndrome (85–87). Mobarrez et al. reports most larger EVs (0.7 - 3.0 μm) found in SLE patients contain functional mitochondrial components, as indicated by the presence of the translocase of outer mitochondrial membrane 20 (TOMM20) and hexokinase1 (25). Elevated levels of these type of EVs containing mitochondria are positively associated with increased SLE disease activity, proinflammatory cytokines, and anti-dsDNA antibodies, suggesting that these EVs may be involved in disease pathogenesis (25). Becker et al. recently reviewed the mechanism of immune activation leading to autoimmune disease by mitochondria in these rheumatic conditions but does not discuss the potential role of viral infections in the process or whether the mitochondrial EVs also contain virus or viral components (85). These findings suggest that damage to mitochondria resulting in autoimmune responses may be a common mechanism in the pathogenesis of many autoimmune diseases.

### Activation of autoimmunity by mitochondrial EVs

One possible mechanism where myocarditis and other autoimmune diseases could be induced and/or exacerbated by mitochondrial EVs is by activation of Toll-like receptor (TLR)4, interleukin (IL)-1β and leucin-rich repeat (LRR)-containing protein (NLRP)3, which is a pathway that has been demonstrated to increase myocarditis and viral replication in CVB3 models of myocarditis (88, 89). Mitochondria are known to be immunogenic and the TLR4/NLRP3 signaling pathway can be activated by mitochondrial components such as cytochrome c, mitochondrial transcription factor A (TFAM), ATP and cardiolipin, which can all be found in mitochondrial EVs, to initiate a proinflammatory and profibrotic immune response (90–94). Mitochondrial and viral antigens may be expressed on the cell surface or interior of EVs and activate APCs via MHC class II presentation, TLRs or be processed for presentation after the EV lipid membrane has ‘merged’ with an APC (23).

However, not all mitochondria found in EVs stimulate the innate immune response. In some cases, healthy mitochondria within EVs are found to fuse with recipient mitochondria in cultured cardiomyocytes and in a mouse model of myocardial infarction where they improve mitochondrial function and disease (95). These investigators showed a similar improvement in doxorubicin-induced toxicity in cultured cardiomyocytes (96). Thus, transfer of healthy intact mitochondria within EVs represents a novel and potentially viable therapy for patients with mitochondrial damage or dysfunction (97, 98).

## Tolerance against mitochondrial antigens and myocarditis

As mentioned earlier, a key feature of the immune response that protects against the development of autoimmunity is the generation of tolerance to self-antigens that occurs in the thymus (1). Since the generation of T cell receptors (TCRs) in the thymus is a random process, negative selection of T cells that react too strongly to self-antigen is required to prevent autoimmunity. To determine whether there are too many self-reactive T cells, the thymus utilizes the autoimmune regulator gene (AIRE) and dendritic cells (99, 100).

### AIRE and tolerance to self

AIRE is a transcriptional regulator that protects against self-reactivity by inducing the production of tissue-specific antigens normally not expressed in the thymus, a process that occurs in medullary thymic epithelial cells (mTECs) (99). Resident dendritic cells of the thymus take up self-proteins and present them to T cells. If reactivity to self-antigen is too strong, dendritic cells undergo cytokine signalling programs that destroy autoreactive T cells (99). Migratory and peripheral dendritic cell populations further contribute to negative selection of autoreactive T cells by selecting against cells reactive to peripheral antigen from other tissue microenvironments. Migratory dendritic cells take up antigen in their respective resident tissues and travel to the thymus whereas peripheral dendritic cells test autoreactive T cells in the periphery (i.e., their tissue of origin) thereby inducing, depending on the conditions, cell deletion, anergy or polarization toward a regulatory phenotype (100). It is estimated that there are 1,140 murine genes that interact with or localize to mitochondrial compartments (101), so AIRE should protect the host from developing autoimmunity against these mitochondrial antigens. One important question is whether mitochondrial genes represent a gap in the normal negative selection criteria in the thymus.

### AIRE and mitochondrial autoimmunity

To our knowledge, no studies examining the function of AIRE describe its ability to produce mitochondrial self-antigen. Two major studies exist with publically available data of AIRE genomic binding and related expression (102, 103). A study by Bansal et al. reported murine transcriptomic data that examined AIRE binding using chromatin immunoprecipitation (ChIP) sequencing (ChIPseq). We examined whether any of the antigens that they reported for AIRE were directed against mitochondrial antigens using their published transcript-level data. They did not have protein/proteomic level data available to assess this question. They estimated AIREs coverage of genes by assessing transcription among AIRE knockout (KO) mice versus wild type (WT) controls in data derived from mouse mTECs (103). A negative log fold-change (LogFC) indicated downregulation of the transcript in the AIRE KO mice, suggesting that in WT conditions, AIRE may be responsible, in part, for regulating the transcription of a respective gene. To determine AIRE regulation of mitochondrial related genes, we performed a keyword search for “mitochondri” (which yielded results for *mitochondrial, mitochondria, and mitochondrion*) in the gene description column of their dataset, which yielded 315 genes. Among these, 20 were significantly downregulated at an FDR *p* ≤ 0.05 comparing AIRE WT to KO indicating that AIRE may regulate only 6.3% of the 315 mitochondrial related genes. We also assessed the potential role of AIRE to regulate 85 murine nuclear-encoded mitochondrial respiratory chain genes using keyword searches in the gene name column for “nduf,” “sdh,” “cox,” “uqcc,” and “atp,” which are the prefixes for gene names among components of respiratory complexes I-V, respectively. Their data showed a significant downregulation of 2 of 85 (2.3%) nuclear encoded mitochondrial respiratory chain genes in AIRE KO vs WT samples. Mitochondrial genes that were regulated by AIRE are listed in **Table 1**. Thus, only a small percentage of potential mitochondrial genes were regulated by AIRE using this method. More research is needed to better understand whether AIRE contributes to tolerance against mitochondrial antigens. Thus, a lack of mitochondrial tolerance may be one possible explanation for the development of mitochondrial targeted autoimmune responses in myocarditis and other autoimmune diseases.

**Table 1:** Mitochondria related and respiratory complex genes expressed by AIRE in mice from Bansal et al. (103) in order of FDR p value.

Gene Symbol	Nominal p value	FDR p value	LogFC*^a^*	Category
Mrpl13	0.0000163	0.000559	-1.29492	Mito Related
Mrps30	0.0000776	0.00182	-0.85468	Mito Related
Gls2	0.000243	0.00422	-0.87328	Mito Related
Mtarc1	0.000294	0.00486	-0.74663	Mito Related
Slc25a13	0.000842	0.0107	-0.84998	Mito Related
Cox7a2l	0.000932	0.0116	-0.87238	Resp Chain
Tmem243	0.00111	0.0131	-0.68091	Mito Related
Cox17	0.00149	0.0161	-0.67956	Resp Chain
Immp1l	0.00158	0.0167	-0.58226	Mito Related
Mtrf1l	0.00211	0.0208	-0.5228	Mito Related
Mrpl22	0.00219	0.0215	-0.58552	Mito Related
Mtarc2	0.00239	0.0228	-0.54555	Mito Related
Mterf1	0.00289	0.0260	-0.46945	Mito Related
Mrpl47	0.00307	0.0273	-0.52963	Mito Related
Mto1	0.00388	0.0325	-0.45504	Mito Related
Tomm20	0.00433	0.0349	-0.53508	Mito Related
Micu2	0.0049	0.0381	-0.46659	Mito Related
Diablo	0.00498	0.0385	-0.47024	Mito Related
Tk2	0.00512	0.0394	-0.44682	Mito Related
Bcat2	0.0069	0.0489	-0.53663	Mito Related

^*a*^Bcat2, branched chain amino acid transaminase 2; Cox7a2l, cytochrome c oxidase subunit 7A2-like; Cox17, cytochrome c oxidase copper chaperone; Diablo, IAP-binding mitochondrial protein; Gls2, glutaminase 2; Immpl1, inner mitochondrial membrane peptidase subunit 1; LogFC, Log Fold-Change; Micu2, mitochondrial calcium uptake 2; Mito Related, mitochondrial related; Mrpl13, mitochondrial ribosomal protein L13; Mrpl22/47, mitochondrial ribosomal protein L22/47; Mrps30, mitochondrial ribosomal protein S30; Mtarc1/2, mitochondrial amidoxime reducing component 1/2; Mterf1, mitochondrial transcription termination factor 1; Mto1, mitochondrial tRNA translations optimization 1; Mtrf1l, mitochondrial translation release factor 1-like; Resp Chain, respiratory chain; Slc25a13, solute carrier family 25 member 13; Tk2, thymidine kinase 2; Tmem243, transmembrane protein 243; Tomm20, translocase of outer mitochondrial membrane 20.

## Summary

In summary, we propose the following possible scenario for the role of mitochondrial EVs in the induction of autoimmune diseases like myocarditis. The initial infection with virus will activate antiviral TLRs like TLR3, 7, 8, 9 in the first few minutes/hours after infection. The virus will traffic to the mitochondria at the local site of infection and mitochondria within the virus’ favorite cell type/primary tropism to obtain a replicative advantage. The virus will be released from the cell in mitochondrial EVs. Mitochondrial components expressed within or on the surface of the EVs then activate TLR4 on APCs. The presence of virus/viral particles and mitochondrial components together may create a strong ‘adjuvant’ effect to activate the immune response. During the viremic stage of viral replication, which typically occurs in the first few days after viral infection, the virus within EVs can traffic through the bloodstream or lymphatics to the heart where infection of cardiac tissues can occur in a non-viral receptor specific manner via EVs or also with viral receptors if they are present in cardiac tissue. For example, CVB3 may enter cardiac cells via coxsackievirus-adenovirus receptor (CAR) which is expressed in the heart. Release of mitochondrial EVs from mitochondrially rich cardiomyocytes may drive a cardiac-specific autoimmune response because the mitochondrial content in/on EVs may contain heart specific mitochondrial antigens. TLR4 signaling has been found to be an important pathway in the pathogenesis of many autoimmune diseases including myocarditis. Autoantibodies against mitochondrial components are found in patients with many different autoimmune diseases including myocarditis and in viral animal models of myocarditis providing evidence of an autoimmune response against mitochondria. Whether mitochondrial EVs that originate from the heart occur at a sufficient level to activate a cardiac-specific autoimmune response may be one reason why myocarditis occurs only rarely. Defects in AIRE may also confer susceptibility to autoimmune responses against mitochondrial antigens in some patients.

## Conclusions

For decades the question of whether viruses can cause autoimmune disease has lacked a plausible explanation. Evidence exists that viral infections cause myocarditis that is also associated with autoimmune responses against the heart in patients and animal models, yet how viruses could cause autoimmunity in myocarditis is not clear. Recent evidence substantiates that many viruses, and in particular the viruses that are associated with clinical cases of myocarditis, target mitochondria to promote viral replication and to evade the immune response they are ejected from cells within EVs. Often these EVs also contain mitochondrial components. It is known that EVs contain proteins, receptors and other components that identify them as originating from self-tissue. EVs that contain replicative virus and/or virus particles and mitochondrial components may form powerful danger signals to the immune system activating TLR4- a key pathway in the pathogenesis of myocarditis and DCM. Autoantibodies against mitochondrial components and specifically cardiac mitochondria are found in patients with myocarditis and DCM providing insight that viral infections may promote the release of mitochondrial antigens to activate an autoimmune response. Additionally, defects in AIRE may allow heightened self-reactivity against mitochondrial antigens. These mechanisms provide an explanation for how viral infections may initiate or promote autoimmune diseases like myocarditis.

## Author contributions

DD: Conceptualization, Data curation, Formal analysis, Methodology, Visualization, Writing – original draft, Writing – review & editing. DB: Conceptualization, Visualization, Writing – review & editing. EM: Writing – review & editing. JS: Conceptualization, Writing – review & editing. TI: Writing – review & editing. DF: Conceptualization, Funding acquisition, Project administration, Supervision, Writing – original draft, Writing – review & editing.
